# Circadian Clock Gene Bmal1: A Molecular Bridge from AKI to CKD

**DOI:** 10.3390/biom15010077

**Published:** 2025-01-07

**Authors:** Songyuan Yang, Zehua Ye, Lijia Chen, Xiangjun Zhou, Wei Li, Fan Cheng

**Affiliations:** 1 Department of Urology, Renmin Hospital of Wuhan University, Wuhan 430060, China; yangsongyuan1999@gmail.com (S.Y.); yezehua1996@whu.edu.cn (Z.Y.); lijiachen@gmail.com (L.C.); zhouxiangjun@gmail.com (X.Z.); 2 Department of Anesthesiology, Renmin Hospital of Wuhan University, Wuhan 430060, China

**Keywords:** circadian clock, Bmal1, AKI, CKD

## Abstract

Acute kidney injury (AKI) and chronic kidney disease (CKD) represent two frequently observed clinical conditions. AKI is characterized by an abrupt decrease in glomerular filtration rate (GFR), generally associated with elevated serum creatinine (sCr), blood urea nitrogen (BUN), and electrolyte imbalances. This condition usually persists for approximately a week, causing a transient reduction in kidney function. If these abnormalities continue beyond 90 days, the condition is redefined as chronic kidney disease (CKD) or may advance to end-stage renal disease (ESRD). Recent research increasingly indicates that maladaptive repair mechanisms after AKI significantly contribute to the development of CKD. Thus, implementing early interventions to halt the progression from AKI to CKD has the potential to markedly improve patient outcomes. Although considerable research has been conducted, the exact mechanisms linking AKI to CKD are complex, and effective treatments remain limited. Kidney function is influenced by circadian rhythms, with the circadian gene Bmal1 being vital in managing these cycles. Recent research indicates that Bmal1 is significantly involved in the progression of both AKI and CKD. In this study, we conducted a retrospective analysis of Bmal1’s role in AKI and CKD, reviewed recent research advancements, and investigated how Bmal1 influences the pathological mechanisms underlying the progression from AKI to CKD. Additionally, we highlighted gaps in the existing research and examined the potential of Bmal1 as a therapeutic target in kidney disease management. This work aims to provide meaningful insights for future studies on the role of the circadian gene Bmal1 in the transition from AKI to CKD, with the goal of identifying therapeutic approaches to mitigate kidney disease progression.

## 1. Introduction

Acute kidney injury (AKI) is a common and critical health condition characterized by a swift reduction in kidney filtration ability, resulting in significant incidence and mortality rates [1,2,3,4,5]. Studies indicate that 12.2% of hospitalized patients develop AKI, with the proportion being even greater in low- and middle-income regions [6]. As AKI advances, it leads to kidney tissue damage, evident through raised serum creatinine, heightened proteinuria, and decreased urine production. AKI carries a mortality rate of 23.9% among adults, with substantially higher fatality rates in patients needing renal replacement therapy. This condition imposes lasting impacts on both individuals and society [7].

**Figure 1:**
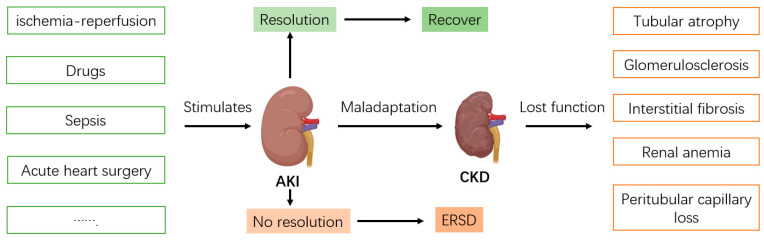
Harmful factors such as infections, nephrotoxic medications, and ischemia–reperfusion can trigger the development of acute kidney injury (AKI). When these harmful conditions persist or intensify, they may lead to cell death, organ failure, and, ultimately, the progression to end-stage renal disease (ESRD). Cellular responses during the repair process play a critical role in patient outcomes. Some cells are capable of repair, regeneration, and recovery; however, others, with poor adaptive responses, may transition from AKI to chronic kidney disease (CKD), eventually leading to ESRD.

Acute kidney injury (AKI) has a multifaceted etiology, with contributing factors such as infections, sepsis, renal ischemia–reperfusion injury, and nephrotoxic medications, each with distinct underlying mechanisms [8,9,10,11,12]. These damaging stimuli activate signal transduction pathways in renal cells, leading to programmed cell death processes like apoptosis and ferroptosis [9,10,11,12,13,14]. After experiencing AKI, patients are at risk of developing chronic kidney disease (CKD), which, in severe cases, can lead to mortality (Figure 1). CKD represents a major global health challenge, impacting roughly 10% of adults. Each year, millions die prematurely due to CKD and its complications, with up to 1.2 million affected annually. As CKD becomes more prevalent, it is projected to rank as the fifth leading cause of death globally by 2040 [15,16,17]. Increasing evidence indicates that AKI serves as an independent risk factor for CKD [18,19,20,21,22,23,24]. Currently, apart from renal replacement therapy, effective treatments for AKI remain limited. The progression from AKI to CKD significantly diminishes patient survival and quality of life.

The circadian regulation of kidney function has recently drawn significant attention for its unique role in the progression from AKI to CKD, especially in relation to tubular cell regeneration and interstitial fibrosis. Among circadian genes, Bmal1 has been the most thoroughly studied concerning kidney injury and fibrosis. Understanding its role could deepen insights into disease mechanisms and aid in developing new preventive approaches.

## 2. Circadian Clock Gene Bmal1

**Figure 2:**
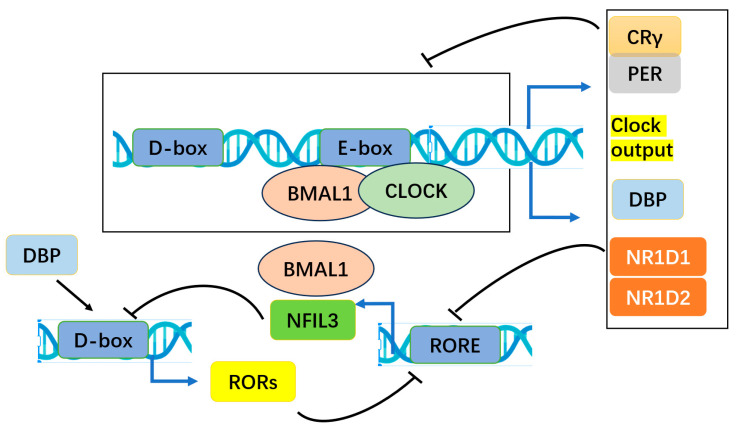
This process illustrates the core clock’s positive feedback loop, where BMAL1 and CLOCK form a heterodimer that binds to E-box elements in the promoters of downstream genes like PER and CRY. The protein products of these genes (PERs and CRYs) establish a negative feedback loop, which diminishes the activity of the BMAL1–CLOCK heterodimer. Additionally, the expression of other regulatory components, such as retinoic acid receptor-related orphan receptors (RORs) and nuclear receptor subfamily 1 group D members (NR1D1 and NR1D2), is modulated through the BMAL1–CLOCK heterodimer’s interaction with E-box elements. This interaction either activates (via RORs) or suppresses (via NR1D1 and NR1D2) BMAL1 expression. Furthermore, protein products from output clock genes, such as NFIL3 and DBP (nuclear factor interleukin-3 regulated NFIL3 and D-box binding protein DBP), affect the regulation of ROR family members and NR1D1/NR1D2, adding another layer of control. This transcriptional feedback loop spans approximately 24 h, governing the circadian rhythmic expression of various genes, commonly known as output clock genes.

Since the 1990s, researchers have identified several genes involved in circadian regulation, including Per, Bmal1, Cry, CLOCK, and Npas2. In 1998, Whitmore D. and colleagues first discovered BMAL1 in fruit flies. Mutations in the CYCLE (CYC) gene, homologous to mammalian Bmal1, have been shown to disrupt normal behavior in fruit flies [25]. BMAL1, a basic helix-loop-helix/Per-ARNT-SIM (bHLH-PAS) transcription factor, partners with CLOCK or NPAS2 to control the transcription of clock-controlled genes (CCGs) via E-box sequences [26,27,28,29,30]. BMAL1 also has a closely related homolog, BMAL2, although BMAL2 has a more limited distribution [31,32,33,34]. In 2010, Shi S. and team uncovered the central role of Bmal1 in circadian clock regulation, leading to its focus in subsequent research [35]. Clock genes are fundamental to transcription–translation feedback loops that generate circadian oscillations. Two core transcription factors, BMAL1 (brain and muscle ARNT-like 1) and CLOCK (circadian locomotor output cycles kaput), are central to the positive feedback loop [36,37,38]. This loop operates through the dimerization of BMAL1 and CLOCK, which bind to E-box elements to drive the transcription of genes that participate in the negative feedback loop, including Period (PER1–3) and Cryptochrome (CRY1 and CRY2). Once translated, PER and CRY proteins are phosphorylated by casein kinase Iε/δ (CKIε/δ) [39,40,41,42] and form a complex that translocates to the nucleus to inhibit the BMAL1-CLOCK dimer, completing the transcription–translation feedback cycle. In addition to initiating the negative feedback loop, BMAL1 and CLOCK bind to E-box elements upstream of retinoic acid receptor-related orphan receptors (RORs) and nuclear receptor subfamily 1 group D genes, NR1D1 and NR1D2 (REVERBA and REVERBB) [43,44], thereby modulating their expression. The resulting proteins compete at ROR response elements on the BMAL1 gene, activating (in the case of ROR) or repressing (in the case of NR1D1 and NR1D2) BMAL1 expression. Additionally, clock output genes, such as NFIL3 (nuclear factor interleukin-3 regulated NFIL3) and DBP (D-box binding protein DBP), regulate the transcription of the ROR (RORA, RORB, RORC) and REVERB (NR1D1, NR1D2) gene families, creating another transcriptional loop that fine-tunes the core clock mechanism (Figure 2). This core regulatory cycle operates over roughly 24 h, establishing the foundation of circadian rhythms [45]. This system also affects the expression of numerous genes, controlling various biological functions. Additionally, circadian rhythms are regulated by post-translational modifications (PTMs), such as phosphorylation, acetylation, and ubiquitination, which affect the stability, localization, and activity of clock proteins. For instance, phosphorylation of PER and CRY proteins, mediated by kinases like casein kinase 1 (CK1), plays a critical role in regulating their degradation and the period of the circadian clock. Moreover, translational regulation also plays a crucial role in circadian rhythm control. Regulatory mechanisms, such as mRNA translation efficiency and the action of specific microRNAs, contribute to the fine-tuning of clock gene expression. These intricate layers of regulation ensure that circadian rhythms remain robust, synchronized with the external environment, and responsive to cellular and environmental cues. BMAL1 is implicated in diverse biological processes and disease pathways. For instance, it is involved in regulating aspects like sleep, body weight, blood pressure, tumor growth, and conditions such as acute kidney injury and chronic kidney disease, as explored in this article [46,47,48,49,50,51].

## 3. Circadian Rhythm in the Kidney

**Figure 3:**
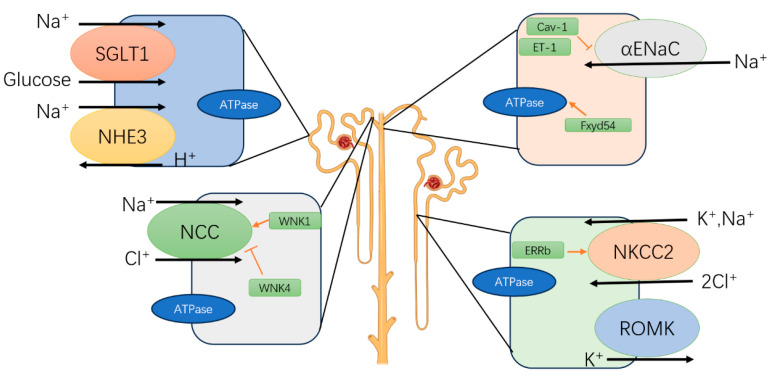
Diagram illustrating nephron structure and various cell types. As blood flows into the nephron, filtration occurs at the glomerulus. The filtrate then enters the proximal tubule (PT), which contains sodium-glucose transporter subtype 1 (SGLT1) and sodium-hydrogen exchanger subtype 3 (NHE3), both regulated by the circadian clock and located on the apical membrane facing the filtrate. Reabsorbed sodium is actively transported back into the bloodstream through the basolateral Na/K-ATPase pump, present across all cell types illustrated here. The filtrate then moves to the loop of Henle, where cells in the thick ascending limb (TAL) express the sodium-potassium-chloride cotransporter subtype 2 (NKCC2), regulated by the nuclear receptor ERRβ. The renal outer medullary potassium channel, ROMK, also operates in these cells to return potassium to the filtrate. TAL cells, like PT cells, contain basolateral Na/K-ATPase. The filtrate next reaches the distal convoluted tubule (DCT), where the apical sodium-chloride cotransporter (NCC) enables sodium and chloride reabsorption from the filtrate, regulated by the circadian clock and WNK kinases. In the nephron’s final segment, the collecting duct (CD), sodium reabsorption is controlled by the epithelial sodium channel (ENaC), also under circadian influence. Additional circadian-regulated proteins, including FXYD5, CAV-1, and ET-1, modulate Na/K-ATPase activity and ENaC function, with FXYD5 enhancing Na/K-ATPase and CAV-1 inhibiting ENaC. Within the juxtaglomerular apparatus, macula densa cells relay information to the glomerulus to adjust the glomerular filtration rate (GFR) through tubuloglomerular feedback (TGF). The glomerulus, PT, and DCT are located in the renal cortex, while the loop of Henle and portions of the collecting duct extend into the medulla. Ultimately, multiple collecting ducts merge to transport urine out of the kidney.

**Figure 4:**
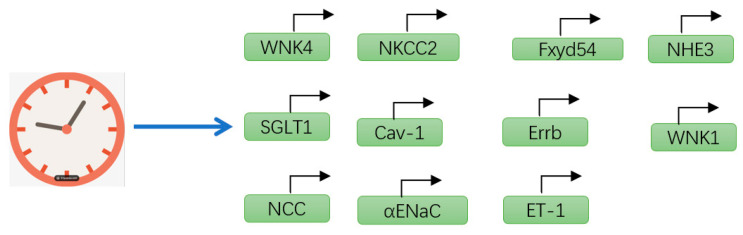
Diagram illustrating that circadian rhythms can regulate the transcription of various proteins associated with kidney function. Sodium-glucose transporter subtype 1: SGLT1; sodium-hydrogen exchanger subtype 3: NHE3; sodium-potassium-chloride cotransporter subtype 2: NKCC2; distal convoluted tubule: DCT; sodium-chloride cotransporter: NCC; collecting duct: CD; epithelial sodium channel: ENaC.

Various kidney functions in humans, such as renal blood flow, glomerular filtration rate (GFR), the corticomedullary osmotic gradient, and tubular transport of water and electrolytes, exhibit circadian rhythms. For instance, Saifur Rohman M. et al. demonstrated that the circadian clock modulates the sodium-hydrogen exchanger (NHE3) and sodium-glucose cotransporter (SGLT1) [52]. Similarly, Pizarro A and Pons M observed that the expression of the sodium-potassium-2 chloride cotransporter (NKCC2) and estrogen-related receptor β (ERRβ) varies in a circadian pattern [53,54]. The α subunit of ENaC (αENaC) and the sodium-chloride cotransporter (NCC) are also subject to circadian regulation [55,56,57,58] (Figure 3). Additionally, Koopman M.G. and colleagues noted circadian fluctuations in glomerular filtration rate (GFR). Specifically, eGFR reaches its minimum value of 86 mL/min around 3:00 AM and its maximum value of 122 mL/min around 2:00 PM, with a relative amplitude of 33% [59]. Meanwhile, Pons M. and team documented distinct circadian rhythms in sodium excretion and renal blood flow [60] (Figure 4).

**Figure 5:**
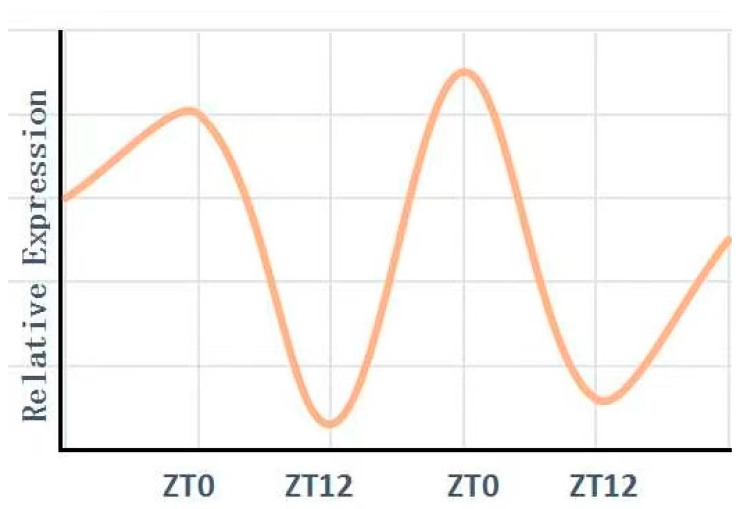
Circadian expression of the core clock gene BMAL1 in the kidney. BMAL1’s relative expression levels are plotted on the *y*-axis, while the zeitgeber time is represented on the *x*-axis. The data shown in this figure were provided by the team led by Pizarro A. [53].

The expression of the clock-regulated gene BMAL1 is tissue-specific [61]. In the kidney, BMAL1 follows a unique circadian rhythm, with expression levels fluctuating over a 48 h cycle (Figure 5) [53]. Firsov and colleagues were the first to knock out the Bmal1 gene specifically in the kidney, creating mice that lacked BMAL1 in renin-producing cells [62]. In these modified mice, BMAL1 expression was absent in juxtaglomerular apparatus cells. Compared to control mice, these Bmal1 knockout mice displayed lower plasma aldosterone levels and a significant drop in blood pressure. Thus, the absence of BMAL1 in certain kidney cells was sufficient to induce systemic changes in both aldosterone and blood pressure. These results highlight the critical role of circadian rhythms and the Bmal1 gene in kidney function regulation.

## 4. Regulatory Role and Mechanisms of Bmal1 in Cell Death During Acute Kidney Injury

Acute kidney injury (AKI) is a major global public health concern, contributing to around 1.7 million deaths annually worldwide. It is also linked to extended hospital stays and significant healthcare costs. One primary characteristic of AKI is the sudden reduction in kidney function [63,64,65,66]. While the pathophysiology of AKI is still not fully understood and requires more research, it is evident that AKI causes damage and death to tubular epithelial cells, with proximal tubular cells being especially susceptible [67,68,69]. In cases where the injury is severe or prolonged, it often leads to maladaptive repair mechanisms, resulting in tubular degeneration, inflammation, renal fibrosis, and eventually progressing to chronic kidney disease (CKD) or end-stage renal disease (ESRD) [70,71,72,73,74].

Research on the circadian rhythm gene Bmal1 in acute kidney injury (AKI) is comprehensive, addressing various causes including AKI resulting from COVID-19, sepsis, ischemia–reperfusion (IR), and drug exposure. Nonetheless, the impact of circadian rhythms and the regulatory mechanisms governing the Bmal1 gene have not been thoroughly investigated. For example, Mercan M. et al. demonstrated that patients with COVID-19-induced AKI experience disruptions in circadian rhythms, which lead to the downregulation of the Bmal1 gene and worsen AKI severity [75]. However, follow-up experiments to clarify the underlying mechanism were not conducted. In an interesting study, Dong C. and his team developed a model of AKI by inducing ischemia–reperfusion (IR) following renal denervation. They observed that denervation interferes with the circadian rhythms of clock genes such as Bmal1, resulting in exacerbation of AKI at specific time points. Their findings indicate that neural innervation influences the rhythmic expression of the Bmal1 gene in the kidney, suggesting that modulation of circadian rhythms can impact AKI prognosis [76]. Our prior research revealed that the Bmal1 gene plays a protective role in models of sepsis-induced AKI. However, the influence of circadian rhythm on sepsis-related AKI is minimal [77]. Similarly, Ye P. et al. found that Bmal1 is involved in maintaining mitochondrial homeostasis during renal ischemia–reperfusion injury (IRI) in mice via the SIRT1/PGC-1α axis, thus providing a protective effect [78]. In a study by Guan S, it was discovered that in a model of AKI induced by 3-monochloro-1,2-propanediol (3-MCPD), 3-MCPD inhibits the SIRT3/SOD2 pathway by altering the rhythmic expression of the Bmal1 gene, leading to mitochondrial damage in the kidney [79]. Additionally, Yoshioka H. et al. reported that the bromobenzene metabolite 4-bromo-o-cresol (4-BrCA) exhibited time-dependent nephrotoxicity; specifically, nephrotoxicity induced by 4-BrCA was observed during the dark phase but not the light phase in mice, which was related to the circadian expression of the Bmal1 gene [80]. In the context of AAI-induced renal injury, Wang Y. and colleagues found that REV-ERBα was upregulated in the kidneys of mice with AAI nephropathy, while its target BMAL1 was downregulated. However, they did not further investigate the role and mechanism of Bmal1 in this process [81]. In contrast, in a cisplatin-induced acute kidney injury model, Zha M. et al. found that Bmal1 enhanced apoptosis and promoted cisplatin-induced renal injury in both in vivo and in vitro studies. Furthermore, circadian rhythms were shown to influence cisplatin-induced acute kidney injury, potentially due to fluctuations in Bmal1 expression [82].

In summary, the function of the circadian rhythm gene Bmal1 varies among different acute kidney injury (AKI) models, sometimes exhibiting opposing effects in these situations. For instance, in the cisplatin-induced AKI model, it worsens renal injury [82], whereas it reduces injury in the LPS-induced AKI model [77]. This variation may result from the specific mechanisms through which different substances interact with cells, underscoring the need for more in-depth research into how these substances influence Bmal1 regulation (Table 1).

## 5. Regulatory Role and Mechanisms of Bmal1 in Chronic Kidney Disease

Late-stage chronic kidney disease (CKD) is a systemic disorder characterized by high mortality rates and diminished quality of life. To prevent progression to renal failure and fibrosis, it is essential to implement various treatment strategies and lifestyle changes. Advancing to later stages of CKD necessitates renal replacement therapy, such as maintenance dialysis or transplantation, which imposes a considerable burden on both patients and society [83,84,85,86,87,88]. The progression of CKD is significantly influenced by hemodynamic and metabolic factors that operate independently of the primary kidney disease. Many of these factors, including blood pressure (BP) and electrolyte disturbances, are impacted by the expression of circadian rhythm genes [53,89,90,91]. Consequently, targeting the circadian rhythm gene Bmal1 may offer a promising therapeutic strategy for individuals with CKD.

Current investigations into the effects of the circadian gene Bmal1 on chronic kidney disease (CKD) primarily focus on directly altering Bmal1 expression to assess its role in chronic kidney injury. For instance, Chen W. and his team discovered that BMAL1 expression levels in kidneys affected by the UUO model were elevated compared to those in normal kidneys, and they noted a significant anti-fibrotic effect associated with Bmal1. Their research identified the ERK1/2/ELK-1/Egr-1 signaling pathway as a critical mechanism for the regulation of BMAL1 [92], although they did not explore how circadian rhythms might influence kidney fibrosis. Similarly, Zhang J. et al. studied Bmal1’s role within the UUO model, observing a reduction in fibrosis following the knockout of Bmal1. They suggested that this reduction could be linked to alterations in the rhythmic expression of Bmal1 after unilateral ureteral obstruction, and that BMAL1 protects against obstructive renal fibrosis by suppressing Gli2 transcription [93]. Additionally, Chen W.D. et al. examined the contributions of the circadian genes Bmal1 and Clock in UUO-induced fibrosis, finding that knocking out the Clock gene modified the circadian expression of TGF-β, thereby impacting fibrosis; however, they did not investigate the specific role of Bmal1 in this context [94]. Interestingly, Rey-Serra C. et al. also researched clock regulatory genes in the UUO model and discovered that the Bmal1 gene was upregulated. However, their findings indicated that knocking out the renal Bmal1 gene had negligible effects on renal fibrosis. They concluded that two other clock genes, Cry1 and Cry2, played a more significant regulatory role in fibrosis [95]. In the adenine-induced CKD model, Fang Y. et al. found that the knockout of the Bmal1 gene in tubular cells led to an exacerbation of renal fibrosis. Interestingly, time-restricted feeding (TRF) in CKD mice partially restored disrupted oscillations of renal clock genes and resulted in improvements in cell cycle arrest and inflammation, leading to reduced fibrosis. This presents new insights into targeting the Bmal1 gene for the treatment of chronic fibrosis [96]. In a model of kidney injury induced by a high-fat diet (HFD), Xing L. et al. observed that constant exposure to light aggravated renal damage, which was related to Bmal1. However, they did not conduct an in-depth investigation into how Bmal1 regulates this process [97]. Liu C. et al. selectively knocked out Bmal1 expression in proximal tubular cells in mice, finding that this knockout worsened adenine diet-induced CKD. The underlying mechanism involved the inhibition of cystathionine β-synthase (CBS) transcription and the disruption of glutathione-related metabolic homeostasis [98]. Conversely, Ansermet C. et al. found that knocking out the Bmal1 gene in renal tubules or glomeruli of type 1 diabetic mice did not significantly affect diabetic nephropathy. Instead, the knockout exacerbated hyperglycemia and increased urinary glucose excretion fraction. This discovery suggests that disruption of the renal tubular circadian clock can exacerbate diabetic hyperglycemia by increasing gluconeogenesis in proximal tubules, independent of any direct connection to chronic kidney disease [99]. Crislip G.R. and his team took an alternative approach. Their earlier studies showed that male skeletal muscle-specific BMAL1 inducible knockout (iMS-BMAL1 KO) mice developed an aging-like profile, marked by gait changes, reduced activity, muscle weakness, and diminished glucose uptake. Considering the possible association between this aging phenotype and chronic kidney disease, further investigation is justified [100].

The circadian rhythm gene Bmal1 plays a significant role in the regulation of renal fibrosis. It has been established that the expression of Bmal1 in the kidneys affects fibrosis and may serve as a therapeutic strategy to mitigate chronic kidney disease and slow its progression. Additionally, the regulation of circadian rhythms is expected to be a viable and effective approach for treating chronic kidney disease. However, different research teams have arrived at varying conclusions regarding the impact of circadian genes on renal fibrosis within the same model. For instance, Chen W. and Zhang J. found that Bmal1 alleviates renal fibrosis in UUO mouse models; in contrast, Rey-Serra C. observed no significant improvement in fibrosis following the knockout of Bmal1 in the kidneys. Therefore, further research is necessary to clarify these discrepancies.

## 6. Therapeutic Potential for AKI and CKD Targeting Bmal1 Signaling

Research has highlighted the important role of the circadian gene Bmal1 in the progression of renal diseases. In particular, Bmal1 offers protective effects in various cases of drug-induced acute kidney injury, with overexpression of the gene showing potential to mitigate these injuries. In contrast, its role in chronic kidney disease is more complex, as Bmal1 knockout leads to different outcomes for renal fibrosis depending on the specific model. For example, while Bmal1 knockout reduces fibrosis in the UUO model, it may worsen fibrosis in other models. Thus, targeting Bmal1 could represent a new therapeutic avenue for treating renal diseases. However, further in-depth studies are required to fully understand its mechanisms in both acute and chronic kidney conditions.

Regulating gene expression through the use of agonists or antagonists is a common approach in both clinical and research fields, allowing for direct intervention in gene expression to exert therapeutic effects. However, there is currently no research on specific inhibitors or agonists targeting the clock gene BMAL1.

Targeted gene therapy, an advanced approach to disease treatment, involves directly altering a patient’s genome. The key types of targeted gene therapy include gene replacement, gene silencing, gene editing, immunogene therapy, gene vaccines, gene transduction, and antisense therapy [101,102,103,104,105,106,107,108,109,110,111,112,113,114] (Table 2). These technologies are rapidly evolving, opening new possibilities for treating genetic disorders, cancer, and infectious diseases. Each type of therapy works through distinct mechanisms and has specific clinical applications. However, the high cost of such therapies remains a major obstacle, limiting widespread access in the near term. Researchers worldwide are actively seeking more affordable and accessible alternatives.

Due to its unique functions, Bmal1 is a promising candidate for gene therapy. By modulating circadian rhythms to regulate Bmal1 expression, its protective role can be enhanced, making it a valuable target for treating diseases of the urinary system. However, there is a lack of clinical research on how modulating circadian rhythms or directly regulating the BMAL1 gene can alleviate acute kidney injury or fibrosis. Further clinical trials are needed to validate its effectiveness, which is crucial for the prevention and treatment of acute kidney injury and chronic kidney fibrosis, and they hold the potential to become an effective therapeutic strategy.

## 7. Conclusions

In this review, we examined the critical role of the circadian clock gene Bmal1, its influence on kidney function, and how circadian rhythm genes regulate processes such as interstitial fibrosis and inflammation during the progression from AKI to CKD. Additionally, we discussed the therapeutic potential and future prospects of Bmal1 in treating both acute kidney injury and chronic kidney disease. We suggest that Bmal1 may act as a protective factor against maladaptive kidney changes that drive CKD and holds promise as a therapeutic target to prevent disease progression.

Here is a revised version:

While progress has been made, several crucial issues remain unresolved. The most pressing is the uncertainty surrounding the clinical effectiveness of circadian rhythm modulation or targeting the Bmal1 gene. Although encouraging outcomes have been seen in animal models with Bmal1 overexpression, further research is essential to assess its safety and practicality for human application. Presently, research is limited to animal studies, and some debates persist, resulting in insufficient clinical evidence to support circadian rhythm regulation or Bmal1-targeted therapies for acute kidney injury and chronic kidney disease. This underscores the need for more comprehensive studies. Additionally, further exploration into Bmal1’s underlying mechanisms is necessary. Beyond its circadian patterns in renal tubules, studies suggest that Bmal1 oscillations in macrophages also play a role in tubular injury [115]. Future research is expected to investigate how Bmal1 expression in non-tubular cells, such as renal fibroblasts, may influence AKI and CKD. Research has also indicated that denervation reduces Bmal1’s rhythmic expression in the kidney, highlighting the role of neural regulation. Moreover, findings show that Bmal1 is regulated by both neural and humoral factors, particularly as seen in the suprachiasmatic nucleus (SCN) of the hypothalamus, which controls Bmal1 expression in peripheral tissues through neural and endocrine pathways. Specifically, the SCN affects Bmal1 expression via rhythmic ion channel activity, electrical signaling, and the release of neurotransmitters like GABA and glutamate, especially within the central nervous system and peripheral organs [116,117,118]. Future research may further explore whether Bmal1 expression in the kidney is influenced by humoral regulation alongside neural mechanisms.

Despite some concerns, we remain confident that investigating the influence of circadian rhythms and the Bmal1 gene in kidney injury is both essential and valuable. Additionally, we expect that extensive research in this field will shed light on the underlying mechanisms and variability in the progression from AKI to CKD, thereby establishing a basis for preventing disease advancement and identifying effective therapeutic targets.

## Figures and Tables

**Table 1:** The Role and Mechanisms of BMAL1 in Different Models of Kidney Injury.

AKI Models	Protective or Harmful	Mechanism	References
COVID-19-induced AKI model (according to retrospective clinical analysis)	Unclear	Unclear	[75]
Ischemia–reperfusion acute kidney injury model (according to preclinical animal models)	Protective	Nrf2/ARE pathway	[76]
Sepsis-induced AKI model (according to preclinical animal models)	Protective	YAP/ACSL4 pathway	[77]
Ischemia–reperfusion acute kidney injury model (according to preclinical animal models)	Protective	SIRT1/PGC-1α axis	[78]
3-monochloro-1,2-propanediol (3-MCPD)-induced AKI model (according to preclinical animal models)	Protective	SIRT3/SOD2 pathway	[79]
4-bromo-o-cresol (4-BrCA) induced AKI model (according to preclinical animal models)	Unclear	Unclear	[80]
AAI-induced renal injury model (according to preclinical animal models)	Unclear	Unclear	[81]
Cisplatin-induced acute kidney injury model (according to preclinical animal models)	Harmful	Unclear	[82]

**Table 2:** Main therapeutic strategies for targeting specific genes.

Serial Number	Therapeutic Strategy	Overview	Application	Example
1	Gene Replacement Therapy	Replacing a defective or mutated gene in the patient by introducing a functional gene.	Frequently applied in the treatment of genetic diseases, such as Cystic Fibrosis and Duchenne Muscular Dystrophy.	Introducing normal genes into liver cells through adeno-associated virus (AAV) vectors for the treatment of hemophilia [101,102]
2	Gene Silencing	Using RNA interference (RNAi) techniques, such as small interfering RNA (siRNA) or antisense oligonucleotides (ASO), to suppress the expression of harmful genes.	Applied in the treatment of diseases associated with overexpression of genes, such as certain cancers and genetic eye disorders.	siRNA is used to target and suppress the gene expression of HIV, thereby stopping the virus from replicating [103,104]
3	Gene Editing	Utilizing tools like CRISPR-Cas9, TALEN, or ZFN to directly and accurately edit specific gene sequences within the genome.	Applied to correct gene mutations that cause diseases, with wide potential uses, such as in cancer, hereditary diseases, and HIV/AIDS.	The CRISPR-Cas9 technique is applied to correct the defective gene in individuals with sickle cell anemia [105,106]
4	Immunogene Therapy	Using genetic engineering to modify immune cells, enabling them to more efficiently identify and target cancer cells or pathogens.	Mainly applied in cancer therapies, such as CAR-T cell treatment.	In CAR-T cell therapy, the patient’s T cells are altered to express chimeric antigen receptors (CAR), enabling them to identify and destroy specific cancer cells [107,108]
5	Gene Vaccines	Vaccines created through genetic engineering introduce genes that encode antigens, stimulating the immune system to generate specific antibodies and T-cell responses.	Broadly used in infectious disease prevention, such as the COVID-19 mRNA vaccines, with potential applications in cancer vaccines.	The mRNA vaccines created by Pfizer and Moderna work by injecting mRNA encoding the spike (S) protein, prompting the body to produce an immune response [109,110]
6	Gene Transduction Therapy	Employing viral or non-viral vectors to introduce genes into target cells to achieve therapeutic effects.	Extensively applied in different types of gene therapy, such as gene replacement, gene silencing, and gene editing.	Employing an adenovirus vector to introduce the normal RPE65 gene into retinal cells, treating genetic retinal disorders [111,112]
7	Antisense Therapy	Antisense oligonucleotides bind to target mRNA, preventing its translation or promoting its degradation, thus suppressing the production of harmful proteins.	Applied in the treatment of amyotrophic lateral sclerosis (ALS), Duchenne muscular dystrophy, and similar diseases.	Eteplirsen is applied in the treatment of Duchenne muscular dystrophy by using antisense oligonucleotides to modify the mRNA of the DMD gene, restoring the expression of partially functional proteins [113,114]

## Data Availability

All data presented in this study are included within the paper.
